# Single Layer Extended Release Two-in-One Guaifenesin Matrix Tablet: Formulation Method, Optimization, Release Kinetics Evaluation and Its Comparison with Mucinex^®^ Using Box-Behnken Design

**Published:** 2017

**Authors:** Amirhosein Morovati, Alireza Ghaffari, Lale Erfani jabarian, Ali Mehramizi

**Affiliations:** a *Department of Pharmaceutics, Faculty of Pharmacy, Pharmaceutical Sciences Branch, Islamic Azad University (IAUPS), Tehran, Iran.*; b *Department of Industrial and Physical Pharmacy, College of Pharmacy, Purdue University, West Lafayette, Indiana 47907, United States. *; c *Tehran Chemie Pharmaceutical Company, Tehran, Iran. *

**Keywords:** Binary mixture, Bilayer tablets, High dose modified release tablets, Highly water-soluble drug, Poor compressibility

## Abstract

Guaifenesin, a highly water-soluble active (50 mg/mL), classified as a BCS class I drug. Owing to its poor flowability and compressibility, formulating tablets especially high-dose one, may be a challenge. Direct compression may not be feasible. Bilayer tablet technology applied to Mucinex®, endures challenges to deliver a robust formulation. To overcome challenges involved in bilayer-tablet manufacturing and powder compressibility, an optimized single layer tablet prepared by a binary mixture (Two-in-one), mimicking the dual drug release character of Mucinex^®^ was purposed.

A 3-factor, 3-level Box-Behnken design was applied to optimize seven considered dependent variables (Release “%” in 1, 2, 4, 6, 8, 10 and 12 h) regarding different levels of independent one (X_1_: Cetyl alcohol, X_2_: Starch 1500^®^, X_3_: HPMC K100M amounts). Two granule portions were prepared using melt and wet granulations, blended together prior to compression. An optimum formulation was obtained (X_1_: 37.10, X_2_: 2, X_3_: 42.49 mg). Desirability function was 0.616. F2 and f1 between release profiles of Mucinex® and the optimum formulation were 74 and 3, respectively. An n-value of about 0.5 for both optimum and Mucinex® formulations showed diffusion (Fickian) control mechanism. However, HPMC K100M rise in 70 mg accompanied cetyl alcohol rise in 60 mg led to first order kinetic (n = 0.6962). The K values of 1.56 represented an identical burst drug releases. Cetyl alcohol and starch 1500^®^ modulated guaifenesin release from HPMC K100M matrices, while due to their binding properties, improved its poor flowability and compressibility, too.

## Introduction

Guaifenesin, 3-(2-methoxy phenoxy) – 1, 2 propanediol, is an expectorant which increases respiratory tract fluid secretions and helps to loosen phlegm. Guaifenesin is a non-hygroscopic, crystalline powder with a low melting point (78.5-79) ºC. This has a highly water-soluble nature (50 mg/mL), readily absorption from the intestinal tract and metabolite excretion in urine. That is said that it could be classified as a BCS class I drug. Regarding its short half-life of 1h, guaifenesin is a suitable candidate for sustained release preparations (1, 2). Owing to its poor flowability (Carr’s index: 55.3%, Hausner’s ratio: 2.2) and poor compressibility, formulating guaifenesin tablets especially with high dose may be a challenge (2, 3). Direct compression may not be feasible when formulation powder properties are not ideal. It will be more discouraging if high drug loading or slow release rates of freely water-soluble drugs are required at the same time (4). In order to lower the amount of release-controlling materials and other excipients, it is likely to employ a suitable processing technique as granulation (5). Granulation process will improve flow and compression characteristics, reduce segregation, improve content uniformity and eliminate excessive amounts of fine particles (5, 6). Although wet granulation is a common approach, melt granulation is an economical, rapid, single step, one-pot and solvent-free method. In this approach, drug substance is subjected to temperatures at least 20 ºC below its melting point but above melting temperature of binders used. It can be advantageous for extended release formulations requiring high drug loading or using freely water-soluble drugs as guaifenesin (5-7). What is more, developing hydrophilic matrices solely, when high doses of water-soluble drugs are used may be a challenge. This is due to the reduction in polymer “%’’ to prevent larger tablet weight, thereby an inconsistent gel layer is formed (8, 9). Hence, effectiveness of incorporating excipients like cetyl alcohol and starch 1500^®^ on the *in-vitro* release profiles of HPMC K100M matrix tablets of guaifenesin were studied. Developing bilayer tablets are currently considered by several pharmaceutical companies mainly due to patent extension, therapeutic effectiveness, and marketing. One of the key advantages of bilayer tablets is the ability to deliver different release profiles of the same API (Active Pharmaceutical Ingredient) like Mucinex^®^ (600 mg guaifenesin extended-release bilayer tablet) (10-13). However, to reduce capital investment, often existing but modified tablet presses are used to develop and produce such tablets. In order to produce a quality bilayer tablet, in a validated and GMP-way, the selected press should be capable of preventing capping and separation of the two individual layers, providing sufficient tablet hardness, preventing cross-contamination between the two layers, producing a clear visual separation between the two layers, high yield, accurate and individual weight control of the two layers. Above all, using a modified tablet press may not be the best approach to produce a quality bilayer tablet under GMP-conditions, especially when high production output is required, too (13–16). Hence, to overcome these challenges, an economical alternative to highly sophisticated tablet press machines to produce bilayer tablets is to develop a single layer tablet, prepared by a binary mixture (Two-in-one), mimicking the dual drug release character of Mucinex^®^. Response surface methodology was used to optimize seven considered dependent variables (Release “%” in 1, 2, 4, 6, 8, 10 and 12 h) with respect to different levels of independent one (X_1_: Cetyl alcohol, X_2_: Starch 1500^®^, X_3_: HPMC K100M amounts). Target values were determined with reference to patent No.: (US 7 838 032 B2) with a title of ‘Sustained Release of Guaifenesin’ and brand characterization. This was used to find the closest formulation to Mucinex^®^ as a release profile point of view in the studied experimental domain. Monolithic matrix systems still have their own limitations including burst effect and fast initial release rate (17, 18). However, this limitation was exploited to have an initial burst release comparable to IR (immediate release) layer of Mucinex^®^.

## Experimental

*Materials*

Guaifenesin was obtained from Synthokem labs Pvt. Ltd. (Hyderabad, India). Cetyl alcohol was purchased from HSH Chemie GmbH (Hamburg, Germany). HPMC K100M (Methocel^®^ K100M premium USP/EP) and Starch 1500^®^ was from Colorcon Ltd. (Dartford, DA26QD, UK). Avicel PH101^®^, PH102^®^ was from FMC Biopolymer (Philadelphia, PA 19103, USA). Lactose Dcl 15 (Pharmatose Dcl 15) was obtained from (DMV pharma, Veghel, The Netherlands). Aerosil 200^®^ (Evonik Degussa) was purchased from Kanchan Rasayan Supplier (Delhi, India). Crospovidone was obtained from Hefei prote chemical Co., Ltd. (Anhui, China). Mg stearate was from All–Chemie Ltd. (Mount pleasant, South Carolina). All other chemicals and solvents were of analytical reagent grade. The commercial 600 mg guaifenesin extended-release bilayer tablet (Mucinex^®^) Lot No.: W000597027 was used as the reference product. 

*Quality target product profiles (QTPPs); Critical quality attributes (CQAs) and Critical process parameters (CPPs) determination*

The ICH Q8 guideline for industry recommends the defining of the QTPPs as it relates to quality, safety and efficacy. On the other hand, the selection of the CQAs is based on the drug substance, excipients, intermediates, and drug product characteristics. CQAs are typically those aspects, which critically affect the product quality, drug release *etc.*, but in each case, they are derived from the QTPPs (19). Identification of CPPs can lead to process design and understanding (Table 1). Design of experiment (DoE) was performed based on initial target formulation obtained from the initial screening (Table 2).

*Preparation of single layer two-in-one guaifenesin matrix tablet*

In order to formulate single layer guaifenesin matrix tablet using two-in-one formulation method, two different granule sizes were prepared and blended together prior to compression (200 tablets for each trial). Four-hundred mg guaifenesin and weighed additives such as Crospovidone, Avicel PH101^®^, lactose Dcl 15, Aerosil 200^®^ were firstly mixed via geometric dilution (preferred method for uniform mixing when ingredients with varying proportions are included), and mixed thoroughly in a poly bag for 10 min thereafter (Mixture 1). Mixture 1 was passed twice through a 20-mesh size sieve. Cetyl alcohol was accurately weighed and melted at 47-53 ºC (25 ºC below the melting point of API) on heating metal and appropriate amount of mixture 1 added gradually to the melted mass, stirred well manually to mix (melt granulation). These granules were then passed through an 18-mesh size sieve (granule portion 1). Two-hundred mg guaifenesin, weighed Avicel PH102^®^ and starch 1500^®^ were firstly mixed via geometric dilution and mixed thoroughly in a poly bag for 10 min thereafter (Mixture 2). Mixture 2 was passed through a 14-mesh size sieve. Appropriate amount of water (10 mL for 58.482 g mixture) was added, while compressed by hand to form a cohesive mass. This mass was then transferred to a plastic bag, exposed to shear with a cylinder moved in a forward and backward direction for five times and subsequently passed through a 12-mesh size sieve. These granules were moved manually in a circular direction for 5 min. However, suitable conditions are mixing under high impeller and chopper speeds. Finely shaped granules were dried in a hot air oven (LABPRO 101, IndianPharma.in., India) for 30 min at 50 ºC. Granules’ moisture was checked using moisture analyzer (Sartorius MA 150, Data Weighing Systems, Inc., Chicago, US). Dried granules were blended with accurately weighed HPMC K100M in a poly bag for 10 min (granule portion 2). Granule portion 2 was added to granule portion 1, blended well in a poly bag for 10 min. Sifted lubricating agent, Mg stearate, was also added to the mixture, blended for 1 min before compression. 

**Table 1:** The selected QTPPs, CQAs and CPPs, their targets and justifications

**Target**		**Justification**
**QTPP elements**		
Dissolution profile	Initial burst release followed by a sustained release profile up to 12h	Quick mucus relief and improve patient compliance
Production method	Mucinex^®^ : Wet granulation - bilayer tablet	Improve tableting properties of guaifenesin in a simple and efficient way, considering its high drug loading, highly water-soluble nature and poor powder properties - Develop it utilizing a cost effective approach
	Two-in-one matrix : limit wet granulation, using melt granulation technique instead – Single layer tablet	
**CQAs**		
Excipients	Mucinex^®^ : Methocel E10M^®^ (Viscosity: 10000 mPa.s) As a tablet binder and control release agent (12.4%) w/w	
	Carbomer 930p : not dissolved but mainly swell to a remarkable extent. As a control release agent at concentrations of 5.0 - 30% w/w (1.2%) w/w	
	Two-in–one matrix: HPMC K100M (Viscosity: 100000 mPa.s) As a control release agent at levels of 10-80% w/w [high viscosity grade of hypromellose was used in small amounts to prevent larger tablet weight] (3.7-8.5)% w/w	To develop a robust modified release tablet with stable, non-toxic, non-irritating excipients in a minimum amounts to prevent larger tablet weight
	Cetyl alcohol: with an acceptable melting point range for melt granulation regarding m.p of guaifenesin does not become rancid, stable in acids, alkalis, light and air (2.4-7.3)% w/w	
	Starch 1500^®^: as a tablet binder, enhance flow and compression characteristics. stable, non-toxic and non-irritant Tablet binder (wet - granulation): 5-10% Tablet disintegrant: 5-10% (0.2-4.9)% w/w	
Dissolution	Based on constraints defined in Table 3	Reach to similar release profile as Mucinex^®^
**CPPs**		
Granule size	Mucinex^®^: 82% of wet granulated guaifenesin is in sustained release portion 18% of wet granulated guaifenesin is in immediate release portion	Due to its high drug loading, It was divided into two portions. This influences dissolution profile and tableting properties
	Two-in–one matrix: 67% of guaifenesin was melt granulated 33% of guaifenesin was wet granulated	

**Table 2:** Initial target formulation

**Ingredients **	**(mg/tablet)**
Guaifenesin	600
Crospovidone	10
Avicel PH101^®^	15
Lactose Dcl15	5
Aerosil 200^®^	10
Cetyl alcohol	**30**
Avicel PH102^®^	32
Starch 1500^®^	**10**
HPMC K100M	**50**
Mg stearate	8
Total Tablet Weight	**770**

**Table 3:** Considered variables (levels and constraints

**Independent variables**	**Different levels**		
	Low	Medium	High
X_1_: Cetyl alcohol amount (mg)	20	40	60
X_2_: Starch 1500^®^ amount (mg)	2	22	42
X_3_: HPMC K100M amount (mg)	30	50	70

**Table 4:** Considered responses (levels and constraints).

**Dependent variables**	**Considered Constraints **	**US Patent Criteria **
Y_1h_: Guaifenesin release “%” in hour 1	33-48%	NMT (not more than) 48%
Y_2h_: Guaifenesin release “%” in hour 2	41-61%	41-61%
Y_4h_: Guaifenesin release “%” in hour 4	62-72%	ND*
Y_6h_: Guaifenesin release “%” in hour 6	73-85%	73-93%
Y_8h_: Guaifenesin release “%” in hour 8	84-90%	ND
Y_10h_: Guaifenesin release ‘’%’’ in hour 10	89-95%	ND
Y_12h_: Guaifenesin release “%” in hour 12	90-100%	NLT (not less than) 90%

* : Not Defined.

**Table 5:** On trial formulation compositions created by Box-Behnken design.

**Ingredients (mg/tablet)** *									
Guaifenesin	Cross povidone	Avicel PH101^®^	Lactose Dcl15	Aerosil 200^®^	Cetyl alcohol	Avicel PH102^®*^	Starch 1500^®^	HPMC K100M	Mg stearate
**F1:** 600	10	15	5	10	20	100	2	50	8
**F2 :** 600	10	15	5	10	60	60	2	50	8
**F3 :** 600	10	15	5	10	20	60	42	50	8
**F4 :** 600	10	15	5	10	60	20	42	50	8
**F5 :** 600	10	15	5	10	20	100	22	30	8
**F6 :** 600	10	15	5	10	60	60	22	30	8
**F7 :** 600	10	15	5	10	20	60	22	70	8
**F8 :** 600	10	15	5	10	60	20	22	70	8
**F9 :** 600	10	15	5	10	40	100	2	30	8
**F10 :** 600	10	15	5	10	40	60	42	30	8
**F11 :** 600	10	15	5	10	40	60	2	70	8
**F12 :** 600	10	15	5	10	40	20	42	70	8
**F13** ^*^ ** :** 600	10	15	5	10	40	60	22	50	8

*: Total tablet weight: 820 mg

*: Diluent that weight was adjusted with

* : Center point replicates (F14, F15).

**Table 6:** Physical properties of the optimized formulation (All values are expressed as mean ± SD, n = 20

**Acceptance criteria**		
Average weight (mg)	819.5 ± 5.60	820 ± 41.00
Average hardness (Kp) *	10.59 ± 1.07	ND^*^
Average thickness (mm)	6.13 ± 0.02	ND
Friability test (%)	0.47	NMT* 0.8 %
Granules humidity (%)	1.8	NMT 2%
Assay (%)	98.6	95 - 105 %
Weight variation (%)	1.42	<5

* : Kilogram-force

* : Not Defined

* : Not More Than.

**Table 7:** Physical properties of on trial formulations (All values are expressed as mean ± SD).

**Formulation number**	**Average weight (mg)**	**Average hardness (Kp)**	**Average thickness (mm)**	**Average friability% (mg/mg)**
F1	831.4 ± 6.10	5.1 ± 0.24	6.76 ± 0.05	0.93
F2	815.6 ± 3.30	7.96 ± 0.45	6.54 ± 0.03	0.79
F3	815.82 ± 5.02	6.62 ± 0.50	6.64 ± 0.07	0.86
F4	821 ± 4.11	8.5 ± 0.75	6.46 ± 0.04	0.76
F5	822.7 ± 8.30	5.86 ± 0.49	6.7 ± 0.06	0.89
F6	819.65 ± 8.00	8.13 ± 0.67	6.51 ± 0.04	0.78
F7	827.35 ± 3.23	6.85 ± 0.28	6.6 ± 0.08	0.84
F8	812 ± 4.59	9.65 ± 0.35	6.29 ± 0.02	0.64
F9	823 ± 2.95	5.13 ± 0.22	6.75 ± 0.07	0.93
F10	826.67 ± 3.35	6.54 ± 0.47	6.66 ± 0.06	0.87
F11	829.5 ± 4.68	6.43 ± 0.42	6.68 ± 0.06	0.88
F12	817.3 ± 3.91	9.14 ± 0.73	6.39 ± 0.03	0.72
F13 *	824.7 ± 5.18	7.11 ± 0.52	6.58 ± 0.04	0.82
F14*	826.8 ± 4.22	7.23 ± 0.49	6.56 ± 0.02	0.82
F15 *	822.5 ± 3.35	7.05 ± 0.33	6.59 ± 0.03	0.82

* : F13, F14, F15 have the same formulation composition.

**Table 8:** Response variables for all 15 runs. (All values are expressed as mean ± SD, n = 6

**Standard order**	**Independent variables**			**Dependent variables**						
	**X**_1_ **(mg)**	**X**_2_ **(mg)**	**X**_3_ **(mg)**	**Y**_1h_ ** (%)**	**Y**_2h_ ** (%)**	**Y**_4h_ ** (%)**	**Y**_6h_ ** (%)**	**Y**_8h_ ** (%)**	**Y**_10h_ ** (%)**	**Y**_12h_ ** (%)**
1	20	2	50	27.05±1.1	42.1±1.1	60±1.4	70.8 ± 1.2	77.8 ± 1.6	83 ± 1.0	85.45 ± 1.3
2	60	2	50	26.1 ± 1.2	40.4 ± 1.7	58.75 ± 1.1	70.2 ± 1.3	77.9 ± 1.0	83.2 ± 1.1	86.35 ± 1.1
3	20	42	50	31.05 ± 2.7	44.5 ± 3.1	60.9 ± 3.0	73.6 ± 3.8	81.2 ± 3.5	86.25 ± 3.5	89.2 ± 2.7
4	60	42	50	23.7 ± 0.9	34.6 ± 1.0	50.1 ± 0.9	61.35 ± 1.3	70.2 ± 1.1	76 ± 1.1	80.3 ± 1.4
5	20	22	30	53.1 ± 3.6	77.7 ± 2.4	97.6 ± 1.6	100	100	100	100
6	60	22	30	61.4 ± 3.6	81 ± 2.5	95.1 ± 1.0	98.1 ± 0.5	100	100	100
7	20	22	70	19.6 ± 0.7	31.15 ± 2.3	50 ± 1.2	62.2 ± 1.3	71.3 ± 1.4	77.5 ± 0.8	81.3 ± 2.1
8	60	22	70	12.85 ± 0.6	24.5 ± 0.6	41.1 ± 0.7	52.4 ± 0.9	61.5 ± 0.8	68.8 ± 0.6	72.75 ± 0.7
9	40	2	30	53.8 ± 2.3	77.4 ± 2.0	94 ± 0.5	97.1 ± 0.4	100	100	100
10	40	42	30	71 ± 3.7	90.6 ± 4.6	99.5 ± 0.6	100	100	100	100
11	40	2	70	21.1 ± 0.8	34.95 ± 1.3	52.8 ± 1.5	64.9 ± 0.9	74.2 ± 0.9	80.1 ± 1.0	83.5 ± 0.9
12	40	42	70	17.7 ± 1.0	29.8 ± 1.1	45.4 ± 0.8	56.9 ± 0.8	66.8 ± 0.7	72.6 ± 0.7	76.2 ± 0.5
13	40	22	50	29.4 ± 2.6	42.4 ± 2.7	60.15 ± 3.0	71.9 ± 2.7	79.5 ± 2.6	85.85 ± 2.4	88.9 ± 2.2
14	40	22	50	32.4 ± 1.2	46.4 ± 1.3	64.2 ± 1.6	75.2 ± 1.3	83.4 ± 1.2	89.5 ± 1.0	92.3 ± 0.8
15	40	22	50	26.3 ± 0.8	39 ± 0.9	56.8 ± 1.2	68.5 ± 0.9	75.8 ± 0.8	82.4 ± 0.6	85.8 ± 0.4

**Table 9:** Standardized main effects of the factors on responses *.

	**Standardized main effects (SME)**									
	**X**_1_	**X**_2_	**X**_3_	**X**_1_ ** X**_2_	**X**_1_ ** X**_3_	**X**_2_ ** X**_3_	**X**_1_ ** X**_1_	**X**_2_ ** X**_2_	**X**_3_ ** X**_3_	***P*** **-Value oflack fit**
Y1h (Quadratic model)			18.59			3.22			6.32	
0.0003*	0.49	0.15	<0.0001	0.36	0.07	0.02	0.11	0.62	0.0014	0.4913
Y2h (Quadratic model)			22.62			2.83			8.57	
0.01	0.16	0.63	<0.0001	0.26	0.18	0.04	0.1	0.51	0.0004	0.6719
Y4h (Quadratic model)	3.05		25.64						9.24	
<0.0001	0.03	0.26	<0.0001	0.14	0.29	0.06	0.14	0.74	0.0003	0.8729
Y6h (Quadratic model)	3.49		22.56						6.55	
0.0001	0.02	0.18	<0.0001	0.07	0.17	0.08	0.15	0.63	0.0012	0.8527
Y8h (Quadratic model)	2.64		16.1						4.17	
0.0006	0.046	0.2	<0.0001	0.1	0.14	0.24	0.16	0.81	0.0086	0.8731
Y10h (Linear model)			9.02							
<0.0001	0.12	0.33	<0.0001	ND*	ND	ND	ND	ND	ND	0.5108
Y12h (Linear model)			8.84							
<0.0001	0.12	0.35	<0.0001	ND	ND	ND	ND	ND	ND	0.5467

* : Only the terms with statistical significance are included.

* : *p*-value of Prob > F

* : Not Defined.

**Table 10:** Analysis of variance data for models

**Model equations**	**R** ^2^	**Adjusted R** ^2^	**Signal-to-noise ratio**	**F-value**	**Noise chance of F-value**	**Lack-of-fit F-value**	**Noise chance of lack-of-fit F-value**
Equation 7 (Y_2h_)	0.9918	0.9770	24.870	67.05	0.01%	0.60	67.19%
Equation 8 (Y_4h_)	0.9936	0.9820	26.088	85.83	0.01%	0.23	87.29%
Equation 9 (Y_6h_)	0.9915	0.9762	23.376	64.71	0.01%	0.26	85.27%
Equation 10 (Y_8h_)	0.9835	0.9537	17.192	33.03	0.06%	0.23	87.31%
Equation 11 (Y_10h_)	0.8861	0.8551	14.678	28.54	0.01%	1.29	51.08%
Equation 12 (Y_12h_)	0.8815	0.8492	14.415	27.28	0.01%	1.16	54.67%

**Table 11:** The predicted and observed responses for the optimized formulation (All values are expressed as mean ± SD, n = 6).

	**Y1h**	**Y2h**	**Y4h**	**Y6h**	**Y8h**	**Y10h**	**Y12h**
Predicted	35.4	52	70.9	80.1	86.5	92.2	93.7
Observed	34.9 ± 1.9	49 ± 1.8	66.8 ± 0.9	78.3 ± 0.9	85.3 ± 1.0	89.6 ± 0.9	91.5 ± 0.9
Predicted error (%) *	-1.4	-5.8	-5.8	-2.2	-1.4	-2.8	-2.3

* : Predicted error (%) = (Observed value - predicted value)/predicted value × 100%.

**Table 12:** Regression coefficients of different release kinetic models and diffusional exponents for on trial formulations.

**Formulation code**	**Hixson-Crowell R²**	**Zero Order R² **	**First Order R²**	**Higuchi R²**	**Korsmeyer R²**	**Diffusional Exponent (n)**
Brand (Mucinex^®^)	0.9752	0.9186	0.8461	0.9805	0.9921	0.4189
F1(20:2:50)	0.9548	0.8938	0.7982	0.9681	0.9781	0.4635
F2(60:2:50)	0.9621	0.9065	0.8109	0.975	0.9828	0.4843
F3(20:42:50)	0.9709	0.9138	0.8342	0.9784	0.9896	0.4318
F4(60:42:50)	0.9842	0.9455	0.8604	0.9925	0.9957	0.4988
F6(60:22:30)	0.7944	0.6394	0.6016	0.773	0.8671	0.1878
F7(20:22:70)	0.974	0.9328	0.8298	0.9871	0.9888	0.5836
F8(60:22:70)	0.9739	0.9471	0.8184	0.9932	0.9848	0.6962
F9(40:2:30)	0.7906	0.644	0.5944	0.7765	0.8603	0.2364
F10(40:42:30)	0.6706	0.4973	0.4782	0.6349	0.7614	0.1233
F11(40:2:70)	0.9687	0.9246	0.8185	0.9839	0.9851	0.5564
F12(40:42:70)	0.9767	0.9409	0.8342	0.9904	0.9895	0.5912
F13(40:22:50)	0.9732	0.9209	0.837	0.9822	0.9906	0.4524
Optimum	0.9584	0.8874	0.8119	0.9641	0.9831	0.3958

**Table 13:** The optimized formulation release “%” data (condition a).

**Response variables**	**Mean guaifenesin release “%” ± SD (n = 6)**	** RSD%**
Y_1h_	33.9 ± 1.4	4.0
Y_2h_	47.25 ± 1.25	2.7
Y_4h_	64.2 ± 0.8	1.3
Y_6h_	75.4 ± 1.2	1.6
Y_8h_	81.1 ± 1.2	1.5
Y_10h_	85.4 ± 1.0	1.2
Y_12h_	88.4 ± 1.2	1.4

**Table 14:** The optimized and brand formulations release “%” data (condition b).

**Response variables**	**Mean guaifenesin (Optimized) release “%” ± SD (n = 6)**	**RSD%**	**Mean guaifenesin (Mucinex** ^®^ **) release “%” ± SD (n = 6)**	**RSD%**
Y_1h_	37.6 ± 2.6	7.4	28.9 ± 1.6	5.5
Y_2h_	49.9 ± 3.0	6.3	41.3 ± 2.0	4.8
Y_4h_	65.2 ± 3.5	5.6	59.0 ± 2.9	4.9
Y_6h_	74.6 ± 2.6	3.5	71.4 ± 3.0	4.2
Y_8h_	82.0 ± 2.6	3.2	80.1 ± 2.4	3.0
Y_10h_	87.25 ± 1.72	2.0	86.9 ± 2.2	2.5
Y_12h_	90.2 ± 2.2	2.4	91.2 ± 2.6	2.9

**Table 15:** The optimized and brand formulations release “%” data (condition c).

**Response variables**	**Mean guaifenesin (Optimized) release “%” ± SD (n = 6)**	**RSD%**	**Mean guaifenesin (Mucinex** ^®^ **) release “%” ± SD (n = 6)**	**RSD%**
Y_1h_	35.0 ± 2.8	8.0	32.0 ± 2.4	7.5
Y_2h_	49.2 ± 1.9	3.85	45.9 ± 2.5	5.4
Y_4h_	66.7 ± 2.2	3.3	64.4 ± 2.6	4.0
Y_6h_	76.0 ± 1.2	1.6	76.3 ± 1.9	2.5
Y_8h_	81.9 ± 1.0	1.25	84.7 ± 2.2	2.6
Y_10h_	86.8 ± 1.6	1.8	91.0 ± 2.8	3.1
Y_12h_	90.1 ± 1.6	1.8	94.3 ± 1.7	1.8

**Table 16:** Individual layers and bilayer release %. (All values are expressed as mean ± SD).

**Hour**	**SR layer release (%)**	**IR layer release (%)**	**Bi-layer release (%)**
1	23.1 ± 0.9	12.3 ± 0.1	35.4 ± 1.2
2	35.95 ± 1.0	12.5 ± 0.3	48.45 ± 1.68
4	54.2 ± 1.1	12.5	66.7 ± 2.1
6	67.95 ± 1.0	12.5	80.45 ± 1.31
8	76.2 ± 0.6	12.5	88.7 ± 1.8
10	81.6 ± 0.7	12.5	94.1 ± 1.6
12	85 ± 0.2	12.5	97.5 ± 1.5

**Figure 1:**
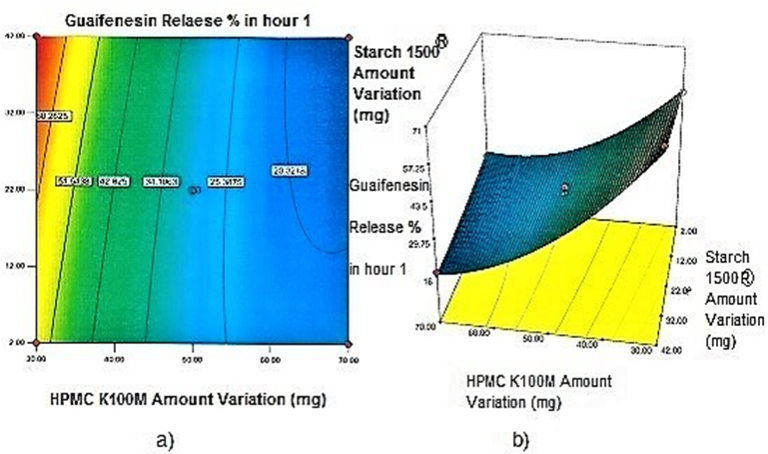
a) Contour plot and b) Response surface plot showing the effect of HPMC K100M (X_3_) and Starch 1500^®^ (X_2_) on Y_1h_

**Figure 2:**
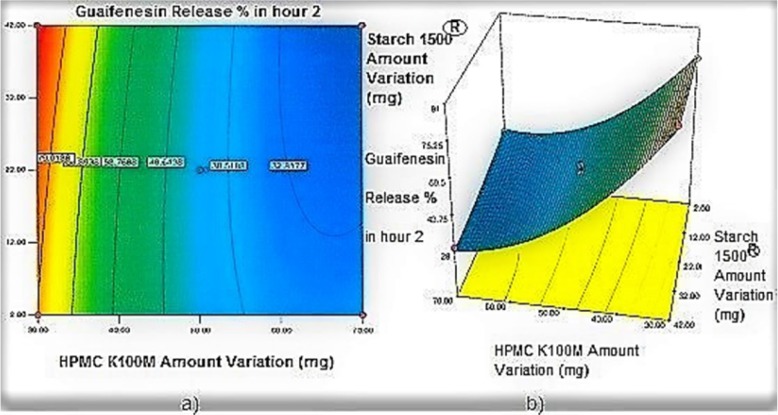
a) Contour plot and b) Response surface plot showing the effect of HPMC K100M (X_3_) and Starch 1500^®^ (X_2_) on Y_2h_

**Figure 3:**
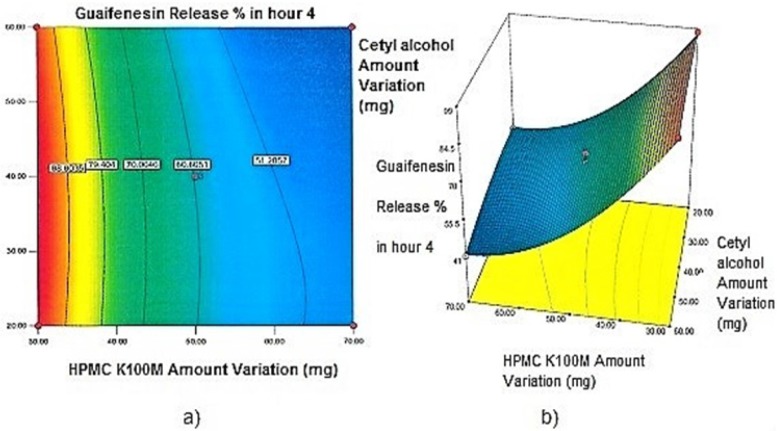
a) Contour plot and b) Response surface plot showing the effect of HPMC K100M (X_3_) and Cetyl alcohol (X_1_) on Y_4h_

**Figure 4:**
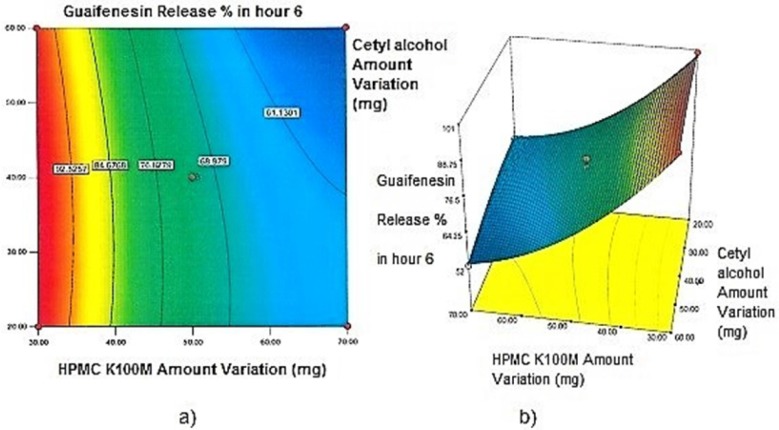
a) Contour plot and b) Response surface plot showing the effect of HPMC K100M (X_3_) and Cetyl alcohol (X_1_) on Y_6h_

**Figure 5:**
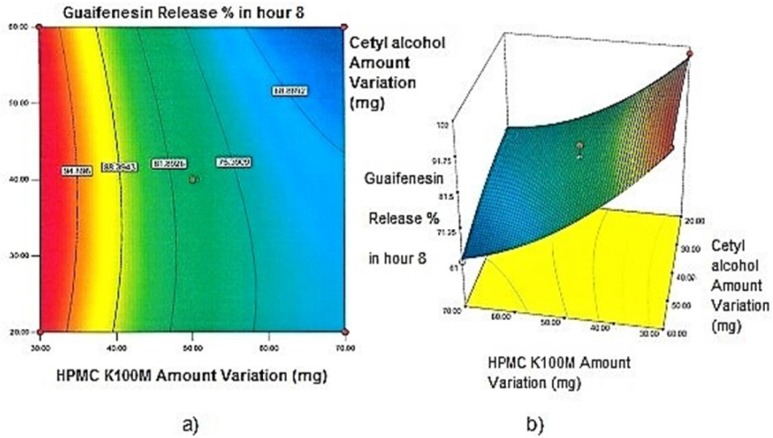
a) Contour plot and b) Response surface plot showing the effect of HPMC K100M (X_3_) and Cetyl alcohol (X_1_) on Y_8h_

**Figure 6:**
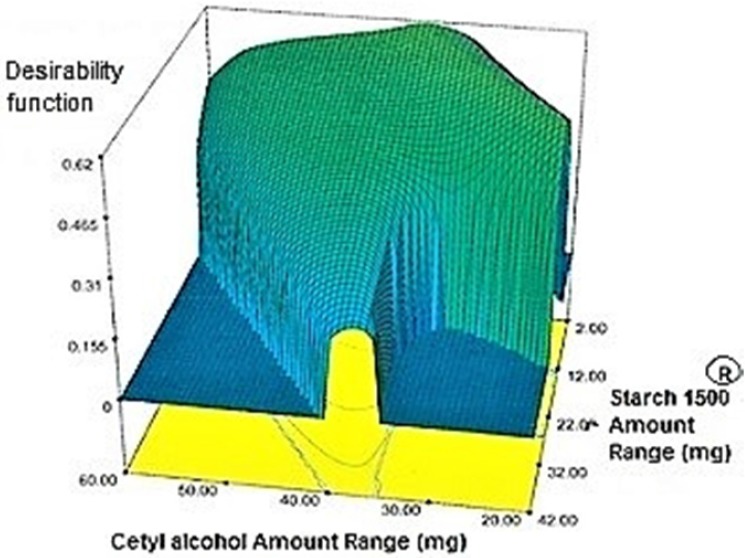
Overall desirability function (D) graph. Starch 1500® amount (X_2_) plotted against Cetyl alcohol amount (X_1_

**Figure 7:**
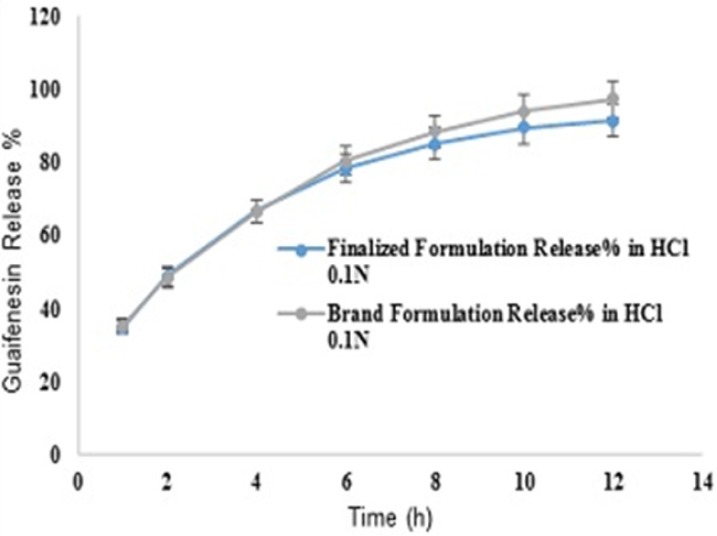
Comparative release profiles of finalized and brand formulations in 900 mL HCl 0.1N (Apparatus I – 75 rpm) [n = 6].

**Figure 8:**
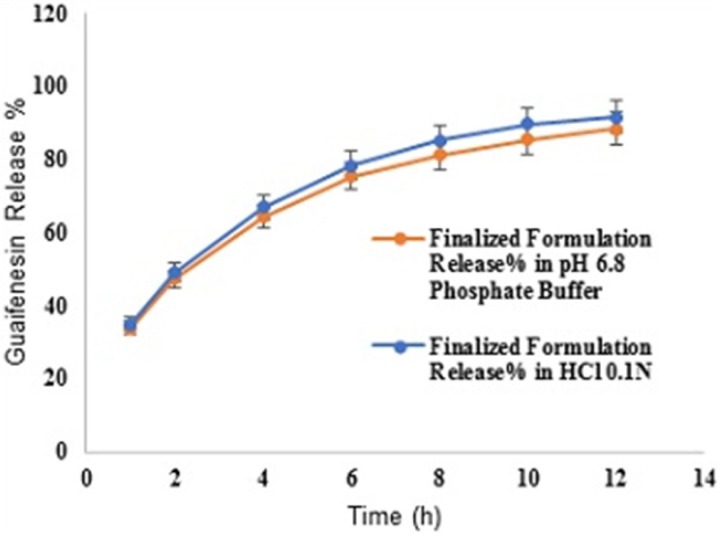
Comparative release profiles of finalized formulation in 900 mL HCl 0.1N and pH 6.8 (PBS) (Apparatus I – 75 rpm) [n = 6].

**Figure 9:**
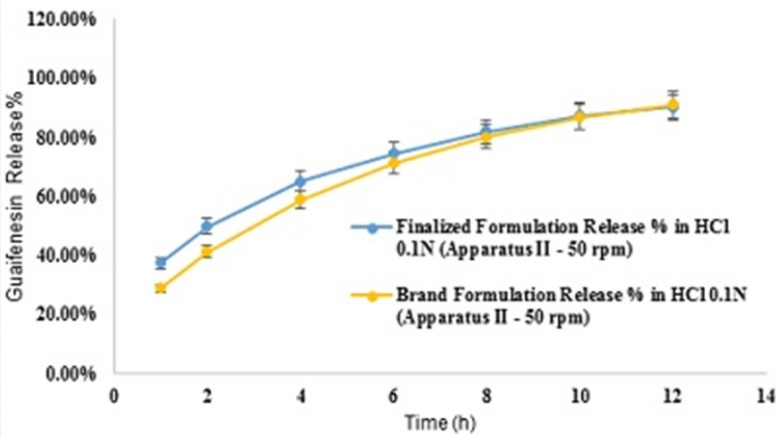
Comparative release profiles of finalized and brand formulations in 900 mL HCl 0.1N (Apparatus II – 50 rpm) [n = 6].

**Figure 10:**
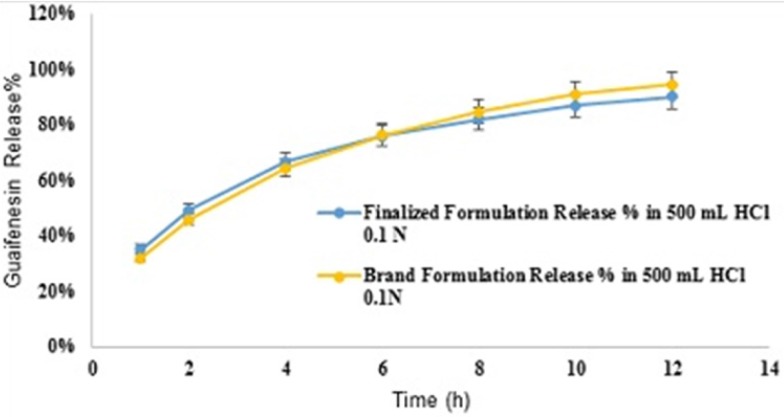
Comparative release profiles of finalized and Brand formulations in 500 mL HCl 0.1N (Apparatus I – 75 rpm) [n = 6].

**Figure 11:**
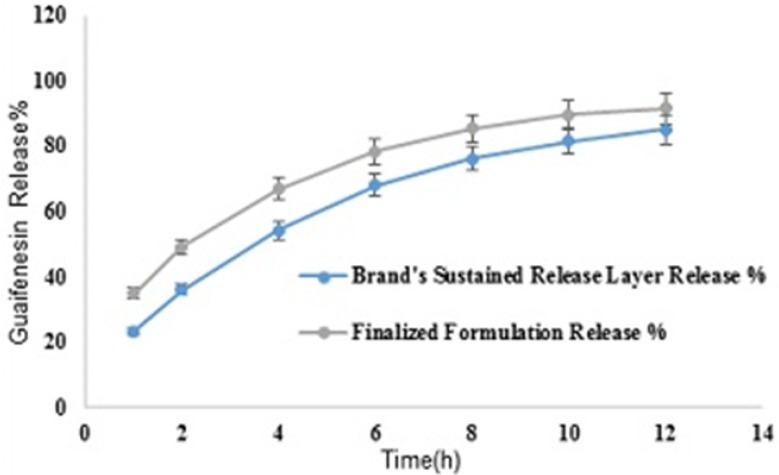
Comparative release profiles of finalized formulation and SR layer of Mucinex® in 900 mL HCl 0.1N (Apparatus I – 75 rpm

**Figure 12:**
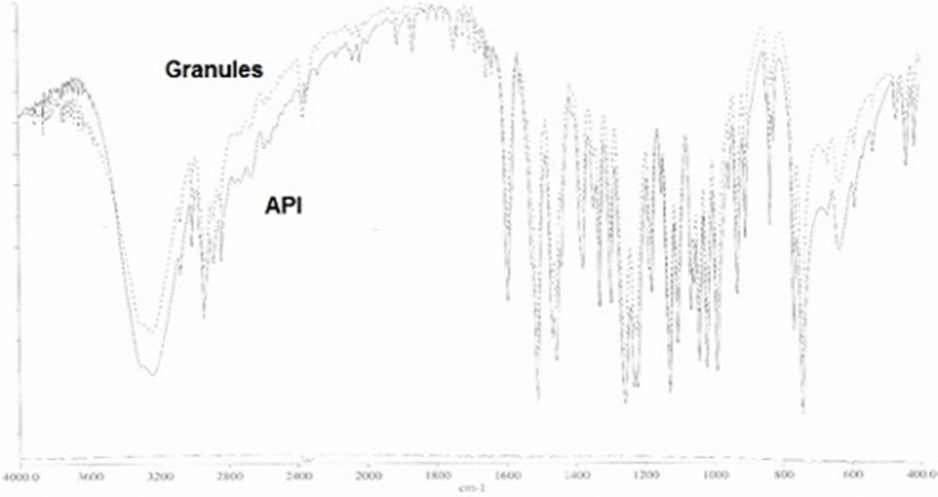
Superimposed FTIR spectra of guaifenesin (API) and granules of the optimized batch

*Experimental design*

Box-Behnken statistical screening design, an economical alternative to Central composite design, was used to optimize and evaluate main effects, interaction and quadratic effects of the independent variables on the *in-vitro* release of single layer two-in-one guaifenesin matrix tablets. A 3-factor, 3-level design used is suitable for exploring quadratic response surfaces and constructing second order polynomial models with Design Expert^®^ (version 7.0.0, Stat-Ease Inc., Minneapolis, MN). This cubic design is characterized by set of points lying at the midpoint of each edge of a multi-dimensional cube and center points replicates (n = 3). The non-linear computer-generated quadratic model is as follows: 

Y = b_0 +_ b_1_X_1 _+ b_2_X_2 _+ b_3_X_3 _+ b_12_X_1_X_2 _+ b_13_X_1_X_3 _+ b_23_X_2_X_3 _+ b_11_X^2^_1 _+ b_22_X^2^_2 + _b_33_X^2^_3_

Where *Y* is the measured response associated with each factor level combination; *b*_0_ is an intercept; *b*_1_ to *b*_33_ are regression coefficients computed from the observed experimental values of *Y*; and *X*_1_*, X*_2_ and *X*_3_ are the coded levels of independent variables. The terms *X*_1_*X*_2_ and *X*^2^_i_ (i = 1, 2 or 3) represent the interaction and quadratic terms, respectively (20, 21). Box-Behnken and Face–centered central composite designs provide three levels for each factor but Box-Behnken design requires fewer runs in the three–factor case (15 *vs.* 20 runs). Moreover, Face–centered design gives poor precision for estimating pure quadratic coefficients. The Box–Behnken design is rotatable (or nearly so) but it contains regions of poor prediction quality. Its “missing corners” may be useful when the experimenter should avoid combined factor extremes. With reference to preliminary experimentation, it was probable to reach the targets through medium levels of each factor. Hence, Box–Behnken seems more desirable since there are more points in the middle of the range and they are not as extreme (22). Table 3 and Table 4 show the dependent variables and their criteria according to patent No.: (US 7 838 032 B2) and brand characterization, as well as independent variables with their low, medium and high levels, achieved via preliminary experimentation. Other excipients’ quantities were kept constant, while Avicel PH102^®^ was used as a diluent in a sufficient quantity to maintain a constant tablet weight (Table 5).

*Characterization of single layer two-in-one guaifenesin matrix tablets*

Matrix tablets were obtained using a 14-station rotary tablet press machine (Manesty Betapress, Liverpool, England) with round-shaped concave punches of 13.5 mm diameter. For each batch, 20 tablets were used to calculate average weight using analytical balance (Sartorius TE 214S, Chicago, US). According to USP37 guideline for tablets with unit mass more than 650 mg, 10 tablets’ friability was evaluated by Electrolab type friabilator (EF-2, ATCOMAART, Mumbai, India) for 4 min at the rate of 25 rpm. The hardness of 20 tablets was evaluated using Erweka hardness tester (TBH 325, ERWEKA GmbH, Heusenstamm, Germany). Average thickness of 10 tablets was measured by Mitutoyo digimatic caliper (500, Mitutoyo Corporation, Japan). 

*Assay procedure for the optimum formulation*

Twenty tablets were weighed individually and crushed into a fine powder with a mortar and pestle. Sample and standard solutions were prepared from sample and standard stock solutions with reference to USP 37 official monographs for guaifenesin tablets. HPLC chromatographic system (Younglin Acme 9000, Younglin Lin Instrument Co. Ltd., South Korea) with a control system of Autochro-3000, UV 276 nm detector, 4.6-mm × 25-cm, 10-µm packing L1 column (Nova – Pak; Waters, MA), mobile phase of methanol, glacial acetic acid and water (40:1.5:60), flow rate of 2 mL /min, and injection size of 20 µL were used to analyze standard and sample solutions. Following formula was used to calculate the percentage of guaifenesin in the portion of tablets taken: 

Result: (r_u_/r_s_) × (C_s_/C_u_) × 100 

r_u_: peak response from the sample solution. 

r_s_: peak response from the standard solution. 

C_s_: concentration of USP guaifenesin RS in the standard solution (µg/mL). 

C_u_: nominal concentration of the sample solution (mg/mL) (Table 6).

*In-vitro dissolution studies*

*Dissolution studies of single layer two-in-one guaifenesin matrix tablets*

Six tablets were randomly chosen for each 15 runs obtained from Box-Behnken design to evaluate mean drug release using USP I (Basket type) apparatus (Electrolab TDT-08L, Mumbai, India) with a rotation speed of 75 rpm and 900 mL HCl 0.1N (simulated gastric fluid) as a dissolution medium. Dissolution procedure was carried out at 37 ± 0.5 °C for 12 h (23). Samples were collected at predetermined time intervals (response variables). A 5 mL aliquot of samples were removed, filtered by soft paper, diluted in 1:10 proportion with a fresh dissolution media and assayed spectrophotometrically using an UV spectrophotometer (UV-1700, Shimadzu Corporation, Japan) at 274 nm. Withdrawn volume was replaced with the fresh medium to maintain sink condition. To evaluate the effects of medium pH, dissolution apparatus type and medium volume on the mean drug release “%”; the following conditions were also considered for the optimized formulation (n = 6): 

900 mL pH-6.8 phosphate buffer solution (PBS, simulated intestinal fluid), apparatus I, 75 rpm for 12 h 

900 mL HCl 0.1N, apparatus II (paddle type), 50 rpm for 12 h

500 mL HCl 0.1N, apparatus I, 75 rpm for 12 h 

*Dissolution studies of Mucinex* ^®^

The same dissolution procedure as the optimized formulation was carried out for Mucinex^®^ (n = 6). Moreover, to determine contribution moiety of individual layers to the overall drug release, surface–mounted (n = 9) Mucinex^® ^(on 16.3 mm die with fixing screw using double-faced adhesive tape) were separated from the intermediate line with a cutter. According to Blume *et al.*, (2002) 75% of total tablet weight may be sustained release (SR) and about 25% of each tablet may be IR formulations (24). Hence, three IR layers were placed in each basket apparatus (n = 3), while one SR layer was placed for each three remained basket apparatuses. Dissolution procedure was conducted in a same condition as Mucinex^®^. 

*Release Kinetics model*

To describe release kinetics model, the experimental data were fitted to the Korsmeyer and Peppas, Hixson-Crowell, Higuchi, zero-order and first-order Equations (Equations 1-5, respectively). 

M_t_/M_∞ _= Kt^n                                                                                          ^(1)

M_t_/M_∞ _= 1- (1- K_1_t)^3                                                                     ^ (2)

M_t_/M_∞ _= b + K_2_t^0.5                                                                            ^(3)

M_t_/M_∞ _= a + K_3_t                                                                                  (4)

Ln M_t_/M_∞ _= c + K_4_t                                                                          (5)

In Peppas Equation (Equation 1), M_t_/M_∞_ is the fraction of drug released up to time *t, K *is the kinetic constant which incorporates structural and geometric characteristics of the drug dosage form and *n *is the release exponent indicative of release mechanism. K_1_ incorporates the surface-volume relation. The regression coefficient values (R^2^) and diffusional exponent (n) were used for evaluation of the release mechanism (25, 26). 

*Fourier Transform Infrared spectroscopy (FTIR)*

Infrared spectra of guaifenesin (API) and granules of the optimized batch were recorded and superimposed on one another using KBr disc method on FTIR (1700, Shimadzu Corporation, Japan) with a Perkin Elmer spectroscopy software (version 5.3) to study compatibility of guaifenesin with excipients used in this study. The samples were analyzed in the frequency range between wave numbers 4000 to 400 cm^-1^ at 4 cm^-1^ resolution. Any change in spectrum pattern of drug due to presence of polymers was investigated to identify any chemical interaction.

## Results

*Physical properties of single layer two-in-one matrix tablets*

The physical properties of the prepared tablets are presented in Table 7. The pharmacopeial limit of “%” deviation for tablets higher than 250 mg is ±5%. Calculated average “%” deviation of each formulation was less than 2%. Calculated friability was less than 1% for all 15 formulations.

Physical properties of the optimized formulation are shown in Table 6. According to USP 37 official monographs for guaifenesin tablets, suitability requirements for the assay procedure are resolution not less than 3.0 between guaifenesin and benzoic acid (system suitability indicating factor) and relative standard deviation of not more than 2.5% for standard solution. [Resolution: 13.6779, RSD”%”: 0.54]. Acceptance criteria are based on patent No.: (US 7 838 032 B2). 

*Experimental design*

*Data fitting to the model*

All response variables for different independent variable amounts are given (Table 8). Mathematical relationships in the form of polynomial Equations for the measured responses are given below (only statistically significant (*p* < 0.05) coefficients are included in the Equations). 

Y_1h _(Release% in 1h) = + 29.37 - 21.01 X_3 _-5.15 X_2_X_3_ + 10.65 X^2^_3_                    (6) 

Y_2h _(Release% in 2 h) = + 42.60 - 25.79 X_3_ - 4.59 X_2_X_3 _+ 14.39 X^2^_3_                  (7) 

Y_4h_ (Release% in 4 h) = + 60.38 - 2.93 X_1_ - 24.61 X_3_ + 13.03 X^2^_3_                     (8) 

Y_6h _(Release% in 6 h) = + 71.87 - 3.07 X_1_ - 19.85 X_3_ + 8.52 X^2^_3_                           (9) 

Y_8h_ (Release% in 8 h) = + 79.57 - 2.59 X_1_ - 15.78 X_3_ + 6.05 X^2^_3_                          (10) 

Y_10h _(Release% in 10 h) = + 85.68 - 12.63 X_3_                                                                         (11) 

Y_12h_ (Release % in 12 h) = + 88.14 - 10.78 X_3_                                                                       (12) 

Positive values indicate a synergistic effect while negative values indicate an antagonistic effect upon the response (27). The above model Equations, in terms of coded factors, can be used to establish the design space. In the most commonly used form of coding, the low, medium and high levels of each factor are coded as -1, 0, +1 respectively. 

*Standardized main effects and reliability of the models*

Table 9 shows the standardized main effects (SME) that were calculated by dividing the coefficient estimate of main effects with its standard error (28, 29). Only statistically significant (*p*-value < 0.05) values are given. Values of prob > F less than 0.0500 indicate that the model terms are significant. Values greater than 0.1000 indicate the model terms are not significant. Furthermore, the *p*-values of lack of fit above 0.05 strengthen the models’ reliability. 

For Equation 6, the R^2^ of 0.9890 is in reasonable agreement with the adjusted R^2^ of 0.9664. Adequate precision measures the signal-to-noise ratio. A ratio greater than 4 is desirable. The ratio of 22.124 indicates an adequate signal. The model F value of 45.71 implied the significance of the model. The chance that a model F value could occur because of noise is 0.03%. The lack-of-fit F value of 1.17 implies the lack of fit is not significant relative to the pure error, and this result is desirable. There is a 49.13% chance that a lack-of-fit F value this large could occur due to noise. ANOVA data for other model Equations are summarized in (Table 10). 

*Contour plots and response surface analysis*

Two-dimensional contour plots, and three-dimensional response surface plots are provided in Figures 1-5, which meet a need to study the interaction effects of the two factors on the response at one time. In all the provided figures, the third factor is kept at a midpoint (zero level). Both the surface and contour plots are based on regression model. Contour and 3D surface plots are useful for establishing response values and operating desirable conditions.

*Optimization*

Target value was determined for each response under the considered constraints defined in (Table 4) by assigning the most importance to responses with the US patent criteria. These targets were 35.4% for Y_1h_, 48.45% for Y_2h_, 66.7% for Y_4h_, 80.45% for Y_6h_, 88.7% for Y_8h_, 94.1% for Y_10h_ and 97.5% for Y_12h_. Individual desirability for each response was calculated and weighed by the importance to which was assigned. These values [1.000 for (Y_1h_), 0.640 (Y_2h_), 0.206 (Y_4h_), 0.952 (Y_6h_), 0.538 (Y_8h_), 0.625 (Y_10h_), 0.491 (Y_12h_)] were combined to determine the composite desirability of this multi-response system. To validate solutions generated by design expert^®^ software, the one with the most composite desirability (0.616) was prepared according to the predicted levels of independent variables (X_1_: 37.10 mg, X_2_: 2 mg, X_3_: 42.49 mg) (Figure 6). As shown in (Table 11), the predicted and observed responses for the optimum formulation reveal no significant difference (*t*-test, *p* > 0.05) and the predicted error “%” are below 6%, indicating that the RSM optimization technique is useful. 

*In-vitro drug release studies*

*Comparison of release profiles using model-independent and dependent methods*

Model independent procedures for comparison of release profiles include the difference factor (f1) and the similarity factor (f2). The difference factor measures the percent error between two curves over all time points: 

F1= (Ʃ^n^_j=1_ ǀ R_j_ - T_j_ ǀ / Ʃ^n^_j=1_ R_j_) × 100 

Where n is sampling number, R_j _and T_j_ are the percent dissolved of the reference and test products at each time point (j). In general, the similarity of dissolution profiles is shown with f1 values lower than 15 (0-15) and f2 values higher than 50 (50-100) (30). Mean guaifenesin release profiles for the optimized and brand formulations (n = 6) in 900 mL HCl 0.1N with f1 and f2 values of 3 and 74 respectively, are depicted (Figure 7). 

The relative standard deviation (RSD) for each dependent variable was less than 10% [Mucinex^®^: RSD(Y_1h_) = 3.5%, (Y_2h_) = 3.5%, (Y_4h_) = 3.2%, (Y_6h_) = 1.6%, (Y_8h_) = 2.1%, (Y_10h_) = 1.7%, (Y_12h_) = 1.5%, Two-in-one: RSD(Y_1h_) = 5.4%, (Y_2h_) = 3.7%, (Y_4h_) = 1.3%, (Y_6h_) = 1.1%, (Y_8h_) = 1.2%, (Y_10h_) = 1.0%, (Y_12h_) = 1.0%].

To assess data fitting of release kinetic model Equations with the same number of parameters, regression coefficient (R^2^) was used (Table 12). The Higuchi and zero-order models represent two limit cases in the transport and drug release phenomena and the Korsmeyer-Peppas model can be a decision parameter between these two models (30). To estimate release controlling excipients percolation thresholds, the Higuchi’s slope was correlated to the predefined levels of independent variables when two out of three variables were at low levels. The third variable was changed to notice slope variations [HPMC K100M amount changes: (20:2:30, K_2_: 12.96), (20:2:50, K_2_: 23.87), (20:2:70, K_2_: 24.36), Cetyl alcohol: (20:2:30, K_2_: 12.96), (40:2:30, K_2_:16.92), (60:2:30, K_2_: 16.66), Starch 1500^®^: (20:2:30, K_2_: 12.96), (20:22:30, K_2_: 16.88), (20:42:30, K_2_: 5.54).

*Different dissolution conditions*

Mean guaifenesin release “%” from the optimized formulation in pH 6.8-phosphate buffer solution (condition a) are summarized in (Table 13). F2 and f1 values between dissolution profiles of the optimized formulation in pH 1.2 and 6.8 were 75 and 4, respectively (Figure 8). Mean guaifenesin release “%” from the optimized and brand formulations in condition b are displayed (Table 14). F2 and f1 values were 63 and 6, respectively (Figure 9). Mean guaifenesin release “%” from the optimized and brand formulations in condition c is given (Table 15). F2 and f1 values were 74 and 4, respectively (Figure 10).

*Brand’s individual layers’ release profile vs. optimum formulation*

Mean guaifenesin release “%” from individual layers of Mucinex^®^ are summarized in (Table 16). F2 and f1 values between dissolution profiles of brand’s SR layer and optimum formulation were 49 and 17, respectively (Figure 11).

*FTIR study*

The superimposed FTIR spectra of API (guaifenesin) and granules of the optimized formulation (guaifenesin + excipients) are shown with two strong peaks representing guaifenesin characteristic bonds (O—H bond, strong broad peaks, 3200-3600 cm^-1^) and (C═O, strong peaks, 1650-1850 cm^-1^) (Figure 12).

## Discussion

Regarding time factor, single layer tablet production increases its capacity with at least 60-70% as compared to bilayer one. The advantage of binary mixtures is that both intimately mixed granular portions interact with each other at a particle/particle level, while in bilayer structure two granule portions only interact at their interface. That is why weak mechanical strength of bilayer tablets is of a great concern, especially when a modified tablet press is used instead of highly sophisticated one. This can lead to enormous financial losses especially when costly drugs are involved (31). Intimate mixing of granules in binary mixtures can be measured by checking fluctuations in tablets’ weight and hardness. Furthermore, uniform distribution of lubricant, which will affect efficient movement of granules into dies, can be considered as an intimate mixing index. The average drug loading capacity reported for conventional dry or wet granulated tablets is usually 50% or less. By contrast, it is reported 85% theoretically and up to 66% actually for melt granulation technique (5, 32). Rise in cetyl alcohol amount (20 to 60 mg) led to highly compactible, stiffer tablets with lower friability. Furthermore, increased HPMC K100M loading (30 to 70 mg) as well as starch 1500^®^ (2 to 42 mg) contributed to this phenomenon. With reference to F1 and F2 formulation contents and physical properties, it is evident that reduction in Avicel PH102^®^ (100 to 60 mg) seems to have no significant effect on hardness of prepared tablets (Table 5, Table 7). In addition, preliminary studies revealed no significant effect on drug release, too (data not shown). Ideal powder flow properties and content uniformity of the optimized formulation were verified by an acceptable tablet weight variation and assay (Table 6). Melt granulation was previously employed to produce tablets with higher hardness and lower friability than that of wet granulation (5). Likewise, melt granulation technology was applied to improve tableting properties of poorly compactible metformin HCl at high dose (33). Extra granular HPMC addition to acetaminophen matrices was reported to increase its hardness (34). Slight compression force increment for the optimized formulation provided stiffer tablets with lower friability and thickness (Table 6). Although tablets became stiffer, no significant difference in release profile was observed when compared with the predicted one (Table 11). The finding complies with what several authors have stated in which the compression force is a statistically significant factor regarding tablet hardness, but its effect on drug release from HPMC tablets was found to be minimal (35, 36). By contrast, Crowley *et al.* (2004) reported that guaifenesin release rate decreased with increasing compaction force in ethyl cellulose matrix tablets prepared by direct compression owing to greater densification of the powder bed (37). Mathematical relationships revealed that X_3 _(HPMC K100M) was an overriding factor in all response variables. X_3_ had the main effect, which means greater change in responses caused by varying X_3_ levels (Equations 6-12). The larger SME values of X_3_ strengthened this importance (Table 9). Two interactions were found between X_2_ (starch 1500^®^) and X_3_ in Y_1h_ and Y_2h_. The interactions precluded drug release in hours 1 and 2. X_1_ (cetyl alcohol) had its own retarding effect on responses Y_4h_, Y_6h _and Y_8h_ with no interaction. X_3_ alone, governed drug release in hours 10 and 12. Combination of fatty acids, alcohols like cetyl alcohol or waxes at low concentrations (≤ 7.5% w/w) with HPMC reported possibility in attaining the extended release of metformin, a highly water soluble active (38). In one study, incorporation of starch 1500^®^ in HPMC matrix tablets caused slower drug release via forming an integral structure within HPMC gel layer (39). By contrast, the super disintegrant prejel^®^ (starch 1500^®^) significantly affected initial water uptake by HPMC tablets of acetaminophen (40). Figures 1a and 2a represent somewhat linear increasing trends toward release retardation with augmentation of HPMC K100M and starch 1500^®^ amounts. On the other hand, Figures 3a, 4a and 5a depict somewhat linear increasing trends toward higher drug release with lowering the amount of HPMC K100M or increasing cetyl alcohol quantities (40 to 60 mg). Response surface plots represent these findings in a 3D graphical representation. The slope of shifts in guaifenesin release “%” due to quantity variations in HPMC K100M and starch 1500^®^ seems to be higher for Y_2h_ in comparison with Y_1h_ (Figures 1b and 2b). Relatively larger regression coefficient of X^2^_3_ for Y_2h_ in relation to Y_1h_ strengthens this finding (Equations 6 and 7). As the color gets darker (blue), guaifenesin release “%” decreases. Comparing 3D surface plots of Y_4h_, Y_6h_ and Y_8h_, it is evident that darker regions are becoming limited when this period (4-8 h) is passing. Reduced regression coefficient of X_3_ (overriding factor) in Equations 8-10, supports this evidence (Figures 3b to 5b). Composite desirability (D) graph shows limited number of combinations among cetyl alcohol and starch 1500^®^ levels (green regions in design space) to reach target values for all the responses. In contrast, there is a large zone in which D is zero (dark blue regions) (Figure 6). The optimum formulation obtained out of a feasible factor space region, represented a similar release profile as Mucinex^®^ (Figure 7). Release profile of the optimum formulation was not affected by the changes in pH of the medium. Figure 8 depicts faster release of guaifenesin in acid medium (HCl 0.1N), which was not significant and reported for guaifenesin tablets containing Carbopol^®^ 971P NF polymer, too (41). HPMC polymers are non-ionic; thereby minimize interaction problems when used in acidic, basic or other electrolytic systems (42). Cetyl alcohol is chemically inert and insoluble in water. Hence, these attributes impart pH change insensitivity and safe application in human to cetyl alcohol as well as HPMC polymers (4, 6). However, according to FDA, an approved maximum potency levels of HPMC K100M and cetyl alcohol in oral extended release formulations are 480 mg and 59 mg, respectively (43). The optimized formulation and Mucinex^®^ release profiles were not significantly affected by dissolution apparatus type and medium volume change, represented condition independent dissolution (Figures 9 and 10). SR layer of Mucinex^®^ did not show any similarity in release profile to the optimum formulation. Interestingly, the burst effect appeared in optimum formulation with no predetermined IR layer (Figure 11). When HPMC (especially high-viscosity grade) matrices of highly water-soluble drugs *e.g. *guaifenesin undergo hydration to form a protective gel layer (lag time), an initial burst release may occur. This phenomenon may be ascribed to the rapid dissolution of the drug from the surface and near the surface of the matrix (39, 44). As seen in formulations with HPMC K100M at 70 mg, swelling was not sufficient to cause complete gelation; therefore, interior of the tablets formed a dry core. Hence, an incomplete drug release was observed within 12 h. In contrast, formulations with HPMC K100M at 30 mg with respect to cetyl alcohol and starch 1500^®^ quantities, released the entire drug within 4-6 h (Table 8). This was ascribed to thinner gel layer formed. Crowley *et al.* (2004) reported ethyl cellulose matrix tablets of guaifenesin with sustained release of 6-8 h (37). Mean guaifenesin release “%” obtained for IR layer of Mucinex^®^ confirmed its complete dissolution within 1 h (45). The average sum of individual layers’ release “%” were equivalent to bilayer tablet in predetermined time points (Table 16). Regarding R^2^ values of different release kinetic Equations, closer to one shows more linearity. This implied Higuchi model for both optimum and Mucinex^®^ formulations (Table 12). However, R^2^ values of Higuchi model showed more linearity in condition b (optimum formulation: 0.9821 *vs.* 0.9641- Mucinex^®^: 0.9900 *vs.* 0.9805). In addition, an n-value of about 0.5 showed diffusion control mechanism. The K values of 1.56 for both the optimum and Mucinex® formulations showed an identical burst drug releases. As reported for diffusional exponent of matrix tablets, an n-value of about 0.5 indicates diffusion control (Fickian diffusion), an n-value of about one denotes erosion or relaxation control (Zero order or type II transport). Intermediate values suggest that diffusion and erosion contribute to the overall release mechanism (non-Fickian or anomalous phenomena, first order kinetic) (46). All formulations, except those with HPMC K100M at 30 mg, showed diffusion (Fickian) release mechanism. However, HPMC K100M rise in 70 mg accompanied cetyl alcohol rise in 60 mg led to first order kinetic (n = 0.6962). Incorporating lipid-based excipients like cetyl alcohol in HPMC matrices shown to reduce water uptake rate, drug dissolution and diffusion front of the matrix (4, 47). In general, for highly water-soluble drugs like guaifenesin, it is possible to achieve release kinetics controlled by diffusion using high viscosity HPMC (39). According to percolation theory, the existence of the critical points where the kinetic properties undergo important changes can be attributed to the modification of the matrix structure close to percolation thresholds (48). Evaluating release profile results as well as release mechanisms indicated the existence of critical points situated between 30 to 50 mg of HPMC K100M, 20 to 40 mg of cetyl alcohol and 42 to 22 mg of starch 1500^® ^related to their percolation thresholds. Above the thresholds, an infinite cluster of components formed which is able to control the hydration and release rate. Below the thresholds, the release controlling agents do not percolate the system and the drug release is not controlled (48). With this in mind, to ensure batch to batch consistency, it would be advisable to use around 50 or 50 to 70 mg HPMC K100M, around 40 mg cetyl alcohol and approximately 22 mg of starch 1500^®^. These quantities were close to the optimum independent variable values determined by design expert^®^. The superimposed FT-IR spectra of guaifenesin and granules of the optimized formulation were fitted well. Although the intensity of granules band reduced, the characteristic peaks of guaifenesin shown indicates absence of any interaction between drug and carrier upon mixing them together (Figure 12). In an industrial scale, high shear granulator, fluidized bed melt granulator, tumbling melt granulator and recently twin-screw extruder, which is favorable for developing high-dose modified release tablets, are used for melt granulation. High shear granulator is a batch process, whereas melt extruder is a continuous process (5). In an industrial scale melt granulation has a few controlling parameters in comparison to wet granulation (49). These parameters in this study might be a good suggestion for future research.

## Conclusions

The optimum single layer two-in-one matrix tablets of guaifenesin showed an identical release profile to Mucinex^®^, that is, a rapid rise followed by an extended release phase. This was done without using a predetermined IR layer. In addition, both showed a diffusion (Fickian) control mechanism. Exploiting HPMC K100M for this highly water-soluble drug gave an initial burst release followed by a sustained release up to 12 h. Mathematical relationships revealed that incorporating cetyl alcohol and starch 1500^®^ modulated drug release profiles of HPMC K100M matrices, by its own and interaction with HPMC K100M, respectively. These excipients also improved poor flowability and compressibility of guaifenesin due to their binding properties via melt and wet granulation techniques, respectively. Hot melt granulation technique employed in two-in-one matrix tablet preparation for 67% of the API in comparison to wet granulation applied in Mucinex®, has advantages for scaling-up and process validation. It is due to only a few controlling parameters. Two-in-one formulation method may be an appropriate choice for high-dose modified release tablets decided to have an immediate release profile at first with poor powder properties, when bilayer manufacturing is not possible. 
